# NEIGHBORHOODS AS A SPACE AND PLACE FOR HEALTHY AGING: NOVEL APPROACHES AND A VIEW OF THE FUTURE

**DOI:** 10.1093/geroni/igad104.0016

**Published:** 2023-12-21

**Authors:** Andrea Rosso, Michelle Carlson, Yvonne Michael

## Abstract

The importance of neighborhood environments for health outcomes has gained increasing recognition in the past two decades. Both space (physical environment) and place (social environment) have implications for health behaviors and risk for acute events and chronic diseases. This symposium brings together a series of talks that address space (walkability, physical activity destinations) and place (racial and socioeconomic polarization) for falls and fear of falling (Pam Dunlap), physical activity behaviors (Pat Donahue), and blood pressure (Hoda Magid). The talks also advance methodology and conceptualization of space and place through use of novel exposure measurements (e.g., Google StreetView audits, spatial polarization) and more nuanced characterization of outcomes (e.g., considering risk stratification and various behavioral aspects of physical activity). Finally, we end with two talks demonstrating ways to advance methods, including the use of longitudinal data to understand long-term neighborhood exposures in relation to outcomes in older adults (Michael Desjardins) and incorporation of novel measures into existing cohorts (Jana Hirsch). These talks leverage data from a diverse range of studies including long-standing cohorts (CHS, MESA), an intervention trial, and VA nursing home data. Together, these talks further our understanding of how neighborhood environments relate to health at older ages and demonstrate the ways in which the field can advance our conceptualization of space and place as both barriers and promoters of health. Yvonne Michael will lead a discussion with the panel and audience on future directions and methodological needs for the field.

